# TIFA: a signaling hub linking inflammation, innate immunity, and human disease

**DOI:** 10.3389/fimmu.2026.1862078

**Published:** 2026-06-08

**Authors:** Yizhuo Fu, Yiting Zhan, Limei Li, Wenzhi Shen, Chunlei Guo

**Affiliations:** 1 Henan International Joint Laboratory of Immunology and Model Animals, Henan Collaborative Innovation Center of Molecular Diagnosis and Laboratory Medicine, School of Medical Technology, Xinxiang Medical University, Xinxiang, China; 2 Precision Medicine Laboratory for Chronic Non-communicable Diseases of Shandong Province, Institute of Precision Medicine, Jining Medical University, Jining, China; 3 Department of Hematology, The Second Affiliated Hospital of Hainan Medical University, Haikou, Hainan, China

**Keywords:** ALPK1-TIFA axis, human diseases, inflammation, NF-κB signaling, therapeutic target, TIFA

## Abstract

The adaptor protein TRAF-interacting protein with a forkhead-associated domain (TIFA) has attracted growing interest because of its role in innate immune and inflammatory signaling. Functionally, TIFA facilitates the oligomerization and ubiquitination of TRAF6, which activates NF-κB-dependent inflammatory pathways. Additionally, the ALPK1-TIFA signaling pathway has been identified as a critical link between sensing microbial metabolites and initiating immune responses. Phosphorylation-dependent oligomerization of TIFA promotes the assembly of higher-order signaling complexes, thereby facilitating the recruitment of TRAF family proteins and the activation of multiple downstream pathways. Increasing evidence shows that TIFA is involved not only in inflammatory signaling but also in immune cell functions, chronic inflammation, tumor-related immune regulation, and blood cancers. Notably, TIFA exhibits context-dependent dual roles, functioning as an oncogenic factor in certain biological contexts while acting as a tumor suppressor in others. Therefore, this review summarizes the structural basis of TIFA activation, its signaling mechanisms, and its emerging roles in human disease, and discusses its therapeutic potential.

## 1 Introduction

TIFA is an adaptor protein that serves as a pivotal hub in innate immune signaling. Its functional core lies in mediating phosphorylation-dependent protein–protein interactions (1). TIFA is currently the smallest known protein containing an FHA domain (2). Upon phosphorylation at specific sites, it undergoes intermolecular recognition, leading to self-assembly and the formation of higher-order oligomeric complexes (3). This oligomerization provides a structural platform for the recruitment and activation of downstream signaling molecules.

The forkhead-associated (FHA) domain typically consists of approximately 80–100 amino acids and is predominantly composed of β-sheet structures (4), enabling specific recognition of phosphothreonine-containing motifs. FHA domains are widely distributed in proteins from both prokaryotic and eukaryotic organisms (5), frequently coexisting with functional modules such as kinase domains and RING finger domains. They participate in diverse biological processes, including the DNA damage response, signal transduction, cell cycle progression, and cell growth (6). Through its FHA domain, TIFA recognizes its own phosphorylation site, thereby mediating intermolecular binding and driving oligomer formation.

As an adaptor molecule, oligomerized TIFA effectively promotes the clustering and activation of Tumor Necrosis Factor Receptor (TRAF) family members (such as TRAF6 or TRAF2). TRAFs are broadly involved in downstream signaling of the tumor necrosis factor receptor (TNFR) and Toll-like receptor (TLR) superfamilies, and participate in inflammatory responses, immune regulation, and bone metabolism by modulating the activity of transcription factors such as NF-κB, c-JUN, and ATF-2.

## 2 Location and structure of TIFA

**Figure 1:**
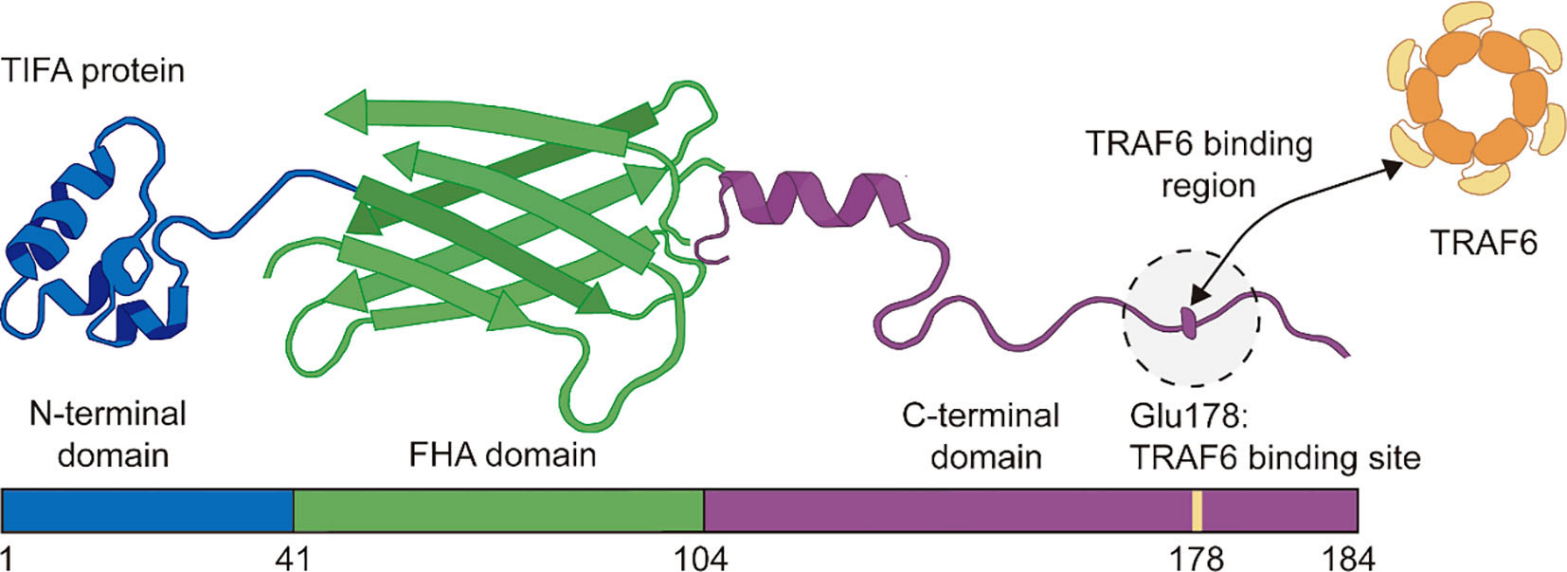
Structural organization of the TIFA protein. A schematic representation of TIFA delineates its domain architecture, comprising the N-terminal domain (residues 1–41), the FHA domain (residues 41–104), and the C-terminal domain (residues 104–184). The TRAF6-binding region is situated within the C-terminal domain, with glutamic acid at position 178 identified as a critical residue mediating the interaction with TRAF6.

TIFA, also known as T2BP, was first identified in a yeast two-hybrid experiment as a binding protein of TRAF6 (7). Subsequently, in mammalian two-hybrid experiments, the same molecule was found to act as a binding protein of TRAF2. Studies have revealed the location and structure of TIFA, which is located on mouse chromosome 3 and human chromosome 4q25. The protein is 184 amino acids in length and consists of three domains: the C-terminal domain, the FHA domain, and the N-terminal domain, with the FHA domain capable of directly binding phosphorylated serine and threonine. In addition to the FHA domain, TIFA contains a specific TRAF6-binding region. Previous studies have demonstrated that the critical binding site resides within the C-terminal domain (8), with glutamic acid at position 178 (Glu178) playing a pivotal role in the interaction with TRAF6 (**Figure 1**).

## 3 Pathways related to TIFA involvement

### 3.1 Intermolecular interaction between TIFA-FHA and TIFA-pT drives TIFA oligomerization

The FHA domain was first described in 1995 and was subsequently reported in 1998 to function as a phospho-protein–binding module (1). FHA domains specifically recognize phosphorylated threonine (pThr, pT) residues and mediate phosphorylation-dependent protein-protein interactions. Although the primary sequences of FHA-containing proteins exhibit relatively low homology, the structural framework of the FHA domain is highly conserved.

Typically comprising 80–100 amino acid residues, the FHA domain adopts a predominantly β-strand configuration. Its core architecture consists of a β-sandwich formed by six- and five-stranded β-sheets, generating a stable and conserved three-dimensional scaffold optimized for phosphothreonine recognition (6). Functionally, FHA-pT interactions regulate a broad spectrum of cellular processes, including DNA damage repair, cell cycle progression, and signal transduction cascades.

**Figure 2:**
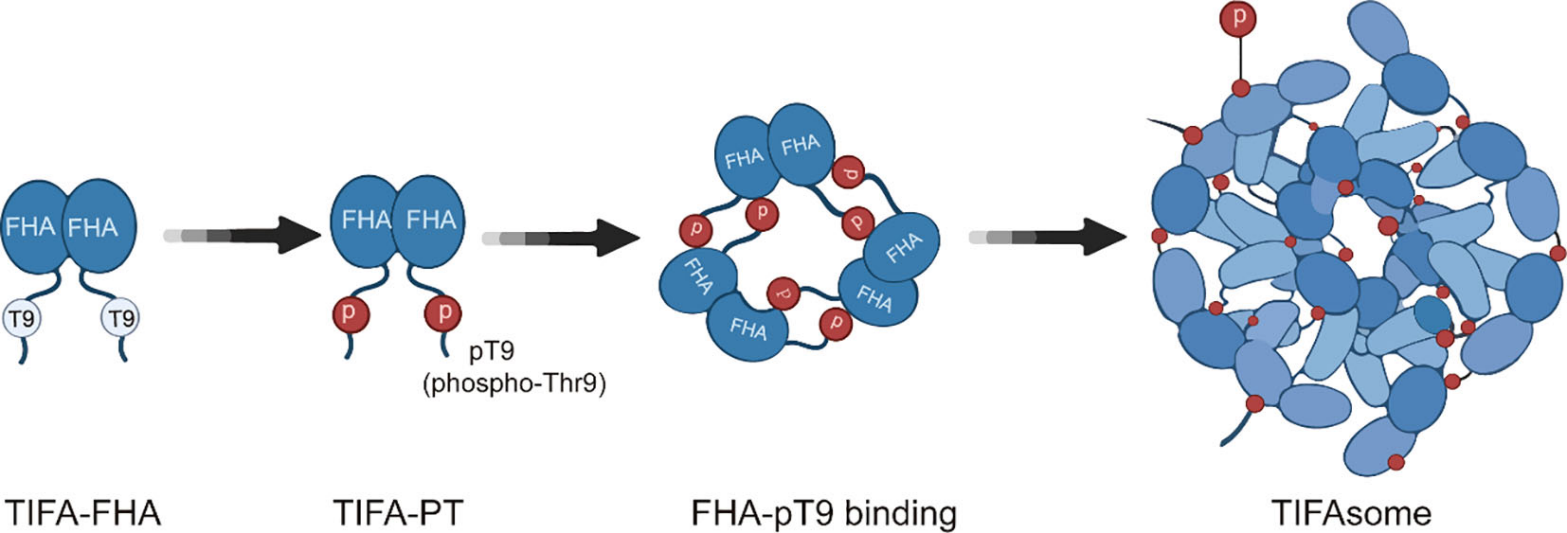
Conceptual model illustrating the assembly of TIFAsomes. Phosphorylation at threonine 9 (Thr9) induces the formation of phosphorylated TIFA (pT9), which is specifically recognized by the forkhead-associated (FHA) domain of neighboring TIFA molecules. The interaction between the FHA domain and pT9 facilitates TIFA oligomerization, thereby promoting the formation of higher-order TIFAsome complexes.

Existing literature has shown that the ninth threonine in the N-terminal domain of TIFA is a phosphorylation site. The FHA domain can specifically recognize this phosphorylation site and bind to it, causing two or even several TIFA proteins to bind together (9), leading to TIFA oligomerization and TIFA-mediated NF-κB activation (**Figure 2**). This intermolecular, phosphate-dependent assembly leads to the formation of higher-order signaling complexes known as “TIFAsomes.” Unphosphorylated TIFA exists in its native dimer form, and the FHA-pT9 interaction occurs between different TIFA dimers (10).

Thr9 is considered a pivotal regulatory residue essential for TIFA function. In a stimulus- and context-dependent manner, TIFA Thr9 phosphorylation can be mediated by distinct upstream kinases: ALPK1 directly phosphorylates TIFA in ADP-heptose sensing (11), Aurora A promotes Thr9 phosphorylation in AML/TNF-α-associated signaling (12), and AKT-associated signaling has also been implicated in TNFR-induced Thr9 phosphorylation (13).

### 3.2 TIFA oligomerization drives TRAF6 activation and amplifies NF-κB signaling

TIFA functions as a critical upstream regulator of TRAF6-mediated NF-κB activation and represents a pivotal node within canonical inflammatory signaling pathways (14). TRAF6 is a RING domain-containing E3 ubiquitin ligase that catalyzes the formation of Lys63-linked (K63) polyubiquitin chains. Unlike Lys48-linked ubiquitination, which targets proteins for proteasomal degradation, K63-linked polyubiquitination primarily serves as a scaffold for signal propagation, facilitating the activation of downstream kinases such as transforming growth factor-β-activated kinase1 (TAK1) and the IκB kinase (IKK) complex (15).

TIFA contains a conserved TRAF6-binding motif, with glutamic acid at position 178 (Glu178) within the C-terminal domain identified. Upon phosphorylation at Thr9, TIFA undergoes FHA domain–dependent oligomerization, leading to the assembly of higher-order TIFAsomes. These TIFAsomes do not directly catalyze ubiquitin chain formation; rather, they function as multivalent signaling platforms that recruit multiple TRAF6 molecules and bring them into close spatial proximity. This TIFA-dependent clustering markedly increases the local concentration of TRAF6 and promotes TRAF6 oligomerization, which is a prerequisite for efficient activation of its RING-dependent E3 ubiquitin ligase activity. (16).

TRAF6 oligomerization is a key step for its E3 ligase activation. Once activated, TRAF6 catalyzes the formation of K63-linked polyubiquitin chains, generating a ubiquitin signaling complex often referred to as TRIKA1. Polyubiquitinated TRAF6 is subsequently recruited to the TRIKA2 complex, which comprises two essential subunits: TAB2, a ubiquitin-binding adaptor protein, and TAK1, a serine/threonine kinase. Recognition of K63-linked ubiquitin chains by TAB2 facilitates TAK1 activation (17).

Activated TAK1 then phosphorylates critical serine residues on the IKKβ subunit within the IKK complex, resulting in IKK activation (18). Active IKK phosphorylates IκB, marking it for degradation and thereby releasing NF-κB from the inhibitory IκB-NF-κB complex. Freed NF-κB translocates from the cytoplasm to the nucleus, where it initiates the transcription of pro-inflammatory genes. Collectively, TIFA-driven TRAF6 oligomerization represents a key amplification mechanism linking upstream phosphorylation events to robust NF-κB activation.

Beyond oligomerization and K63-linked ubiquitin scaffold formation, TRAF proteins are subject to additional layers of post-translational regulation. Notably, Wu et al. identified a previously unrecognized cytoplasmic signaling regulatory network in the context of inflammatory disease, in which SMYD2-mediated lysine methylation of TRAF2 plays a crucial role in sustaining NF-κB activation. SMYD2 specifically methylates TRAF2, a modification that enhances TRAF2 stability by preventing proteolytic degradation and potentiating it signaling activity, thereby maintaining prolonged NF-κB activation. In contrast, the lysine-specific demethylase LSD1 counteracts this modification by removing methyl groups from TRAF2, thus antagonizing SMYD2-dependent signaling (19).

These findings underscore that TRAF-dependent NF-κB signaling is not solely governed by ubiquitin-mediated scaffolding but is dynamically fine-tuned by diverse post-translational modifications, collectively shaping the magnitude and duration of inflammatory responses.

### 3.3 TIFA is involved in the classical TLR/IL-1R/MyD88 signaling pathway

TLR and IL-1R signaling proceed through myeloid differentiation primary response 88 (MyD88) recruitment of interleukin-1 receptor-associated kinase 1 (IRAK) kinases and TRAF6, leading to TAK1 and IKK activation and culminating in NF-κB-mediated inflammatory gene expression (20). Accumulating evidence indicates that TIFA is functionally integrated into the classical TLR/IL-1R/MyD88 signaling pathway. Under these conditions, the interactions among IRAK1, TRAF6, and TIFA is markedly enhanced. Meanwhile, TIFA promotes and stabilizes the interaction between TRAF6 and IRAK1, thereby facilitating downstream NF-κB signal amplification (21).

Mechanistically, TIFA appears to act as a scaffold-like adaptor that reinforces TRAF6-dependent signaling complexes. By strengthening IRAK1-TRAF6 association, TIFA may facilitate TRAF6 activation and subsequent propagation of downstream kinase cascades, including TAK1 and IKK activation. This places TIFA downstream or parallel to the MyD88-IRAK module but upstream of canonical NF-κB activation, allowing it to amplify inflammatory signaling initiated by IL-1R and potentially other MyD88-dependent receptors. In hypoxia–reoxygenation, TIFA upregulation is accompanied by enhanced TLR4/MyD88/NF-κB signaling, further enhancing the release of pro-inflammatory mediators, including high-mobility group box 1 (HMGB1) (22), establishing a positive feedback loop that sustains inflammatory responses (23).

**Figure 3:**
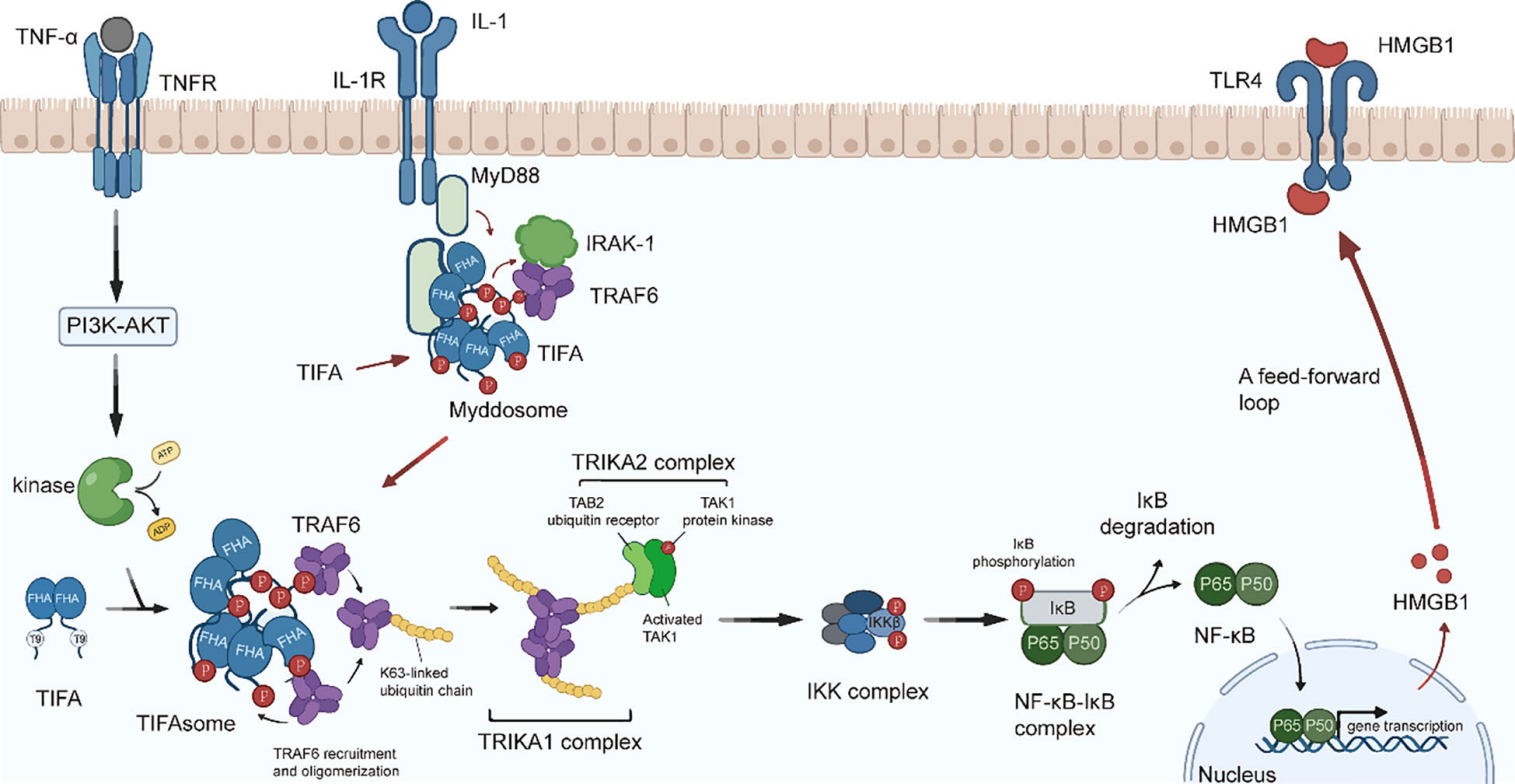
TIFA-mediated signaling facilitates the activation of NF-κB and sustains HMGB1-driven feed-forward inflammatory responses. This schematic depicts the signaling cascade initiated by TIFA downstream of inflammatory receptors. Activation of the tumor necrosis factor receptor (TNFR) triggers the PI3K/AKT pathway, resulting in kinase-dependent phosphorylation of TIFA at threonine 9. Concurrently, interleukin-1 (IL-1) stimulation via the IL-1 receptor (IL-1R) leads to the recruitment of MyD88, IRAK1, and TRAF6 to the myddosome complex, where TIFA is engaged. Phosphorylated TIFA undergoes oligomerization mediated by its FHA domain, forming the TIFAsome complex. This complex facilitates the recruitment and oligomerization of TRAF6, promoting the assembly of K63-linked ubiquitin chains. These molecular events enable the formation of TAK1-containing complexes, which activate the IKK complex, leading to phosphorylation and subsequent degradation of IκB. The degradation of IκB permits the release and nuclear translocation of NF-κB heterodimers (p65/p50), thereby initiating transcriptional activation. Additionally, HMGB1 released downstream can activate Toll-like receptor 4 (TLR4), establishing a feed-forward loop that amplifies inflammatory signaling.

Consequently, elevated TIFA expression strengthens the feed-forward amplification of TLR4/MyD88-dependent signaling, culminating in robust NF-κB activation and HMGB1 release (**Figure 3**).

### 3.4 The ALPK1-TIFA signaling axis expands the functional landscape of TIFA in innate immunity

Early studies identified HBP/ADP-heptose as bacterial cytosolic PAMPs capable of inducing TIFA-dependent NF-κB activation (24). Subsequent work established ALPK1 as the upstream sensor kinase that directly recognizes ADP-β-D-manno-heptose and phosphorylates TIFA at Thr9 (11), thereby triggering TIFA oligomerization and downstream TRAF6/TAK1/NF-κB signaling.

This ALPK1-TIFA axis links bacterial heptose metabolite sensing to innate immune responses in a context-dependent manner. In pancreatic β cells, pro-inflammatory cytokines upregulate ALPK1, enhancing TIFA/TAK1/NF-κB signaling and promoting TNF-α and Fas expression, which increases β-cell susceptibility to inflammatory apoptosis (25). In contrast, in intestinal epithelial cells, ADP-heptose–related metabolites released by Akkermansia muciniphila activate the ALPK1/TIFA/TRAF6 pathway and induce NF-κB-dependent barrier-protective genes, thereby supporting mucosal homeostasis (26).

The ALPK1-TIFA pathway expands the functional role of TIFA in innate immunity by connecting bacterial metabolite recognition with NF-κB-mediated inflammatory or protective responses.

### 3.5 Integration of canonical and non-canonical NF-κB pathway by TIFA

During Helicobacter pylori infection, TIFA contributes not only to the activation of the canonical NF-κB pathway but also plays a pivotal role in regulating the non-canonical NF-κB cascade (27). Through direct interaction with TRAF2, TIFA promotes the dissociation of the TRAF2–cIAP1 complex and facilitates proteasomal degradation of cIAP1. This process stabilizes NF-κB-inducing kinase (NIK), thereby triggering p100 processing into p52 and activating the non-canonical NF-κB signaling axis (28). This mechanism indicates that TIFA can directly affect the non-canonical NF-κB pathway by regulating NIK homeostasis.

Unlike the rapidly inducible canonical NF-κB pathway, the non-canonical pathway is generally slower and depends on NIK stabilization, p100 processing, and RelB/p52 nuclear translocation. Thus, by regulating the TRAF2-cIAP1-NIK axis, TIFA may help shape a delayed but sustained NF-κB response during H. pylori infection, potentially contributing to chronic inflammation, epithelial cell survival, and infection-associated pathological remodeling. This process is also controlled by negative feedback molecules. The classic NF-κB target gene A20 is newly synthesized after infection and interferes with its binding to the NIK regulatory complex through interaction with TIFA, thereby inhibiting alternative NF-κB signaling and anti-apoptotic gene expression, preventing excessive amplification of inflammatory signals (29).

**Figure 4:**
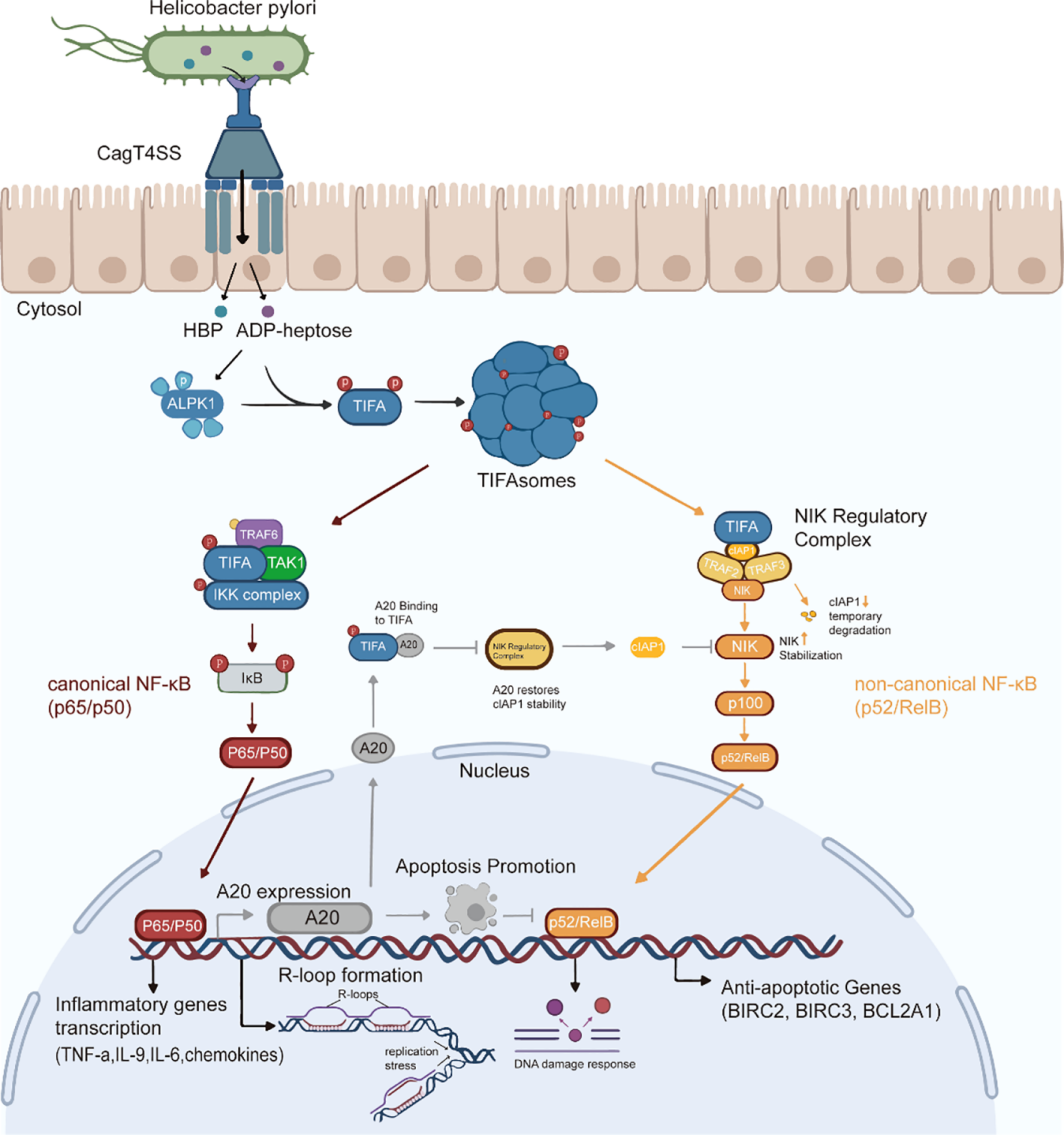
Helicobacter pylori CagT4SS activates TIFA-dependent canonical and non-canonical NF-κB signaling. Schematic model showing that *H. pylori* deliver HBP/ADP-heptose into host epithelial cells through the Cag type IV secretion system (CagT4SS). Cytosolic HBP/ADP-heptose activates ALPK1, leading to phosphorylation of TIFA and formation of TIFAsomes. TIFAsomes recruit downstream signaling components, including TRAF6, TAK1 and the IKK complex, to activate canonical NF-κB signaling and induce p65/p50-dependent inflammatory gene transcription. In parallel, TIFA promotes non-canonical NF-κB signaling through regulation of the NIK regulatory complex, resulting in NIK stabilization, p100 processing, p52/RelB activation and transcription of anti-apoptotic genes. A20 induced by canonical NF-κB binds TIFA and restores cIAP1 stability, thereby limiting NIK activation and providing negative feedback between the two NF-κB pathways. TIFA-dependent signaling further contributes to R-loop formation, DNA damage responses and apoptosis regulation during *H. pylori* infection.

Mechanistically, H. pylori deliver β-ADP-heptose into host cells via its *cag*PAI type IV secretion system, leading to activation of ALPK1 and phosphorylation of TIFA at Thr9 (30). This modification drives TIFAsome assembly. Oligomerized TIFA recruits TRAF6 and promotes its K63-linked polyubiquitination, thereby activating the TAK1–IKK kinase cascade and initiating canonical NF-κB signaling (**Figure 4**). Collectively, TIFA functions as a molecular signaling hub that integrates TRAF2- and TRAF6-dependent pathways, coordinating crosstalk between canonical and non-canonical NF-κB signaling during bacterial infection.

### 3.6 TIFAB as a negative regulator of TIFA-TRAF signaling

TIFAB is an FHA-domain-containing paralog of TIFA and has emerged as an important negative regulator of TIFA-TRAF signaling (2). Canonical TIFA signaling relies on phosphorylation-dependent TIFA oligomerization, TIFAsome formation, recruitment of TRAF6, and subsequent TRAF6 ubiquitination, which collectively promote activation of the TAK1/IKK/NF-κB and MAPK pathways (9). This mechanism is particularly relevant in innate immune sensing of bacterial heptose metabolites, such as ADP-heptose or HBP, through the ALPK1-TIFA axis (3).

In contrast to TIFA, TIFAB lacks or diverges in key elements required for productive TIFAsome-dependent TRAF6 activation, and experimental evidence supports its inhibitory role in limiting TIFA-TRAF6 signaling output (2). Mechanistically, TIFAB may interfere with TIFA-dependent signaling by forming non-productive complexes with TIFA or by restricting TRAF6 recruitment, ubiquitination, and downstream signal amplification.

This negative regulatory function is especially relevant in hematopoietic and inflammatory contexts, where loss or reduced expression of TIFAB, including in chromosome 5q-deleted myeloid malignancies, has been associated with enhanced TRAF6-linked inflammatory signaling and dysregulated NF-κB activation (31).

Therefore, TIFA activity is shaped not only by upstream activators such as ALPK1 and bacterial ADP-heptose/HBP, but also by intrinsic paralogous.

### 3.7 Post-translational control of TIFA-TRAF signaling and stress integration

Beyond activation, TIFA–TRAF signaling is tightly regulated by post-translational mechanisms that determine its strength and duration. Phosphorylation of TIFA, particularly at Thr9, drives TIFAsome assembly (10), whereas TRAF-dependent ubiquitin remodeling shapes downstream NF-κB signaling and may also influence MAPK output (16). In this way, phosphorylation initiates signal propagation, while ubiquitin-based regulation shapes signaling amplitude and persistence.

Negative regulation is equally important for preventing excessive inflammatory activation. Ubiquitin-editing factors such as A20 can terminate signaling by removing or remodeling activating ubiquitin chains (29). In addition, TIFA undergoes stimulus-dependent turnover, which likely represents an active signal-termination mechanism. In epithelial infection models, TIFA is stabilized by proteasome and lysosome inhibitors and associates with trafficking adaptors linked to endolysosomal sorting, suggesting that activated TIFA complexes may be cleared through coordinated proteasomal, lysosomal, and possibly selective autophagy-related pathways (32).

Whether additional regulatory layers, including transcript-level modifications and other stress-responsive mechanisms, modulate the TIFA-TRAF axis remains to be established. More broadly, these post-translational mechanisms may be relevant not only to innate immune signaling but also to cellular stress integration, particularly in conditions such as infection and DNA damage, where inflammatory signaling and ubiquitin-dependent stress responses intersect.

### 3.8 DNA damage and genotoxic stress signaling

TIFA is also involved in NF-κB activation induced by DNA damage, thereby regulating the survival of leukemia cells under genotoxic stress. In cooperation with TRAF2, TIFA promotes ubiquitination of the NF-κB regulatory subunit NEMO, effectively coupling DNA damage signals to NF-κB activation. NEMO ubiquitination and phosphorylation constitute critical steps in this pathway. Following genotoxic stress, TIFA translocated from the cytoplasm to the nucleus, where it accumulates at sites of damaged chromatin and enhances transcription of canonical NF-κB target genes, including IL-6 and IL-8 (33).

However, the upstream kinases, chromatin-associated binding partners, and signal termination mechanisms that regulate TIFA in the DNA damage context remain incompletely defined.

## 4 TIFA in human diseases

### 4.1 Gastric cancer

Chronic Helicobacter pylori infection represents a major etiological risk factor for gastric cancer, primarily through persistent activation of NF-κB signaling and establishment of a chronic inflammatory microenvironment (34). H. pylori subverts host signaling pathways to induce tolerogenic dendritic cells, suppress effector T cell responses, and remodel the gastrointestinal microbiota, thereby driving disease progression from chronic gastritis and peptic ulceration to gastric malignancy (35).

Mechanistically, H. pylori activate NF-κB signaling through delivery of heptose metabolites into gastric epithelial cells via its CagT4SS (36). Then TIFA signaling initiates a cag4 type secretion system-dependent innate immune response to Helicobacter pylori infection (37). Specifically, β-ADP-heptose is sensed by ALPK1, triggering ALPK1 autophosphorylation and subsequent phosphorylation of TIFA at Thr9 (30). This event induces TIFA oligomerization and TIFAsome assembly. The TIFAsome serves as a signaling scaffold that recruits TRAF6 and TRAF2, thereby coordinating activation of both canonical and non-canonical NF-κB pathways (27).

On one hand, the TIFA-TRAF6 axis promotes K63-linked polyubiquitination and activation of the TAK1-IKK kinase cascade, culminating in nuclear translocation of p65/p50 and transcription of pro-inflammatory genes. On the other hand, TIFA interaction with TRAF2 induces degradation of cIAP1, stabilizes NF-κB-inducing kinase (NIK), and promotes p100 processing to p52, thereby initiating non-canonical NF-κB signaling. The dual pathway activation mechanism is the core driving force of the inflammatory response in gastric epithelial cells (38).

Helicobacter pylori infection can induce significant replication stress and DNA double-strand breaks in S-phase gastric epithelial cells. Mechanistically, the bacterial lipopolysaccharide biosynthesis intermediate β-ADP-heptose is recognized by ALPK1, which activates TIFA and initiates the NF-κB signaling pathway, thereby inducing the formation of co-transcriptional RNA/DNA hybrids (R-loops). Bacterial infection triggers TIFA- and NF-κB-driven innate immune responses, linking them to R-loop-dependent replication stress and DNA damage (39).

Beyond direct signaling activation, H. pylori fine-tune inflammatory progression through regulation of the heptose biosynthesis pathway. The key heptose biosynthetic enzyme HldE exhibits marked sequence variability among strains. Its expression is regulated by the cag pathogenicity island, host cell contact, and the carbon storage regulator A (CsrA), collectively determining ADP-heptose output (40). Such strain-specific regulation may modulate the magnitude of ALPK1-TIFA axis activation and thereby influence oncogenic risk.

Faass et al. demonstrated that Neutrophils display a highly sensitive, cell-autonomous response to bacterial heptose intermediates. Exposure to heptose metabolites not only potently activates neutrophils but also reshapes their transcriptional regulatory networks and maturation programs. Activation of human neutrophils by live H. pylori depends on LPS-associated heptose metabolite production and a functional CagT4SS. Similar immune activation effects have been validated in both *in vitro*–differentiated and primary human neutrophils (41). These findings underscore that bacterially derived heptose metabolites trigger strong neutrophil innate responses via the ALPK1-TIFA signaling axis, further amplifying infection-associated inflammatory microenvironments.

Recent biochemical studies further refined this regulatory framework. García-Weber et al. reported that *in vitro* kinase assays ALPK1 undergoes autophosphorylation upon ADP-heptose stimulation, and disease-associated ALPK1 mutants exhibit altered kinase activity (42). In gastric epithelial cells infected with H. pylori, ALPK1-dependent TIFA activation is followed by a reduction in TIFA protein levels, a process blocked by proteasomal and lysosomal inhibitors. Notably, TIFA degradation occurs independently of TRAF2, TRAF6, TAK1, or NEMO. Mechanistically, H. pylori promote TIFA interaction with free polyubiquitin chains and autophagy adaptor proteins, including optineurin, TAX1BP1, and LAMP1, suggesting that activated TIFA is cleared through a ubiquitin-dependent lysosomal pathway (32).

TIFA acts as a key signaling hub in gastric cancer after ALPK1 senses bacterial heptose metabolites, driving the formation and maintenance of a chronic inflammatory microenvironment by integrating the TRAF6-dependent canonical NF-κB pathway with the TRAF2/NIK-mediated non-canonical NF-κB pathway, thereby promoting the occurrence and progression of Helicobacter pylori-associated gastric cancer.

### 4.2 Lung adenocarcinoma

In lung adenocarcinoma (LUAD), TIFA expression is significantly elevated and positively correlates with poor clinical outcomes. In a cohort of 116 LUAD cases, TIFA expression was markedly elevated in tumor tissues compared with adjacent non-malignant counterparts and was significantly correlated with poorer overall survival. Its expression was also closely associated with tumor differentiation and TNM stage, and multivariate analysis identified TIFA positivity as an independent predictor of unfavorable prognosis. *In vitro*, TIFA was highly expressed across multiple LUAD cell lines. Silencing of TIFA significantly inhibited cell proliferation and migration, induced cell cycle arrest and apoptosis (43), upregulated p53, p21, and cleaved caspase-3, and downregulated Bcl-2, Cyclin D1, and CDK4. Moreover, reduced phosphorylation of IKKβ, IκB, and p65 following TIFA knockdown indicated suppression of NF-κB signaling, suggesting that TIFA promotes LUAD progression, at least in part, through activation of this pathway (44).

Collectively, these findings establish TIFA as a promoter of proliferative and migratory phenotypes in LUAD cells. By coordinating cell cycle progression and apoptosis related signaling pathways, at least in part through modulation of NF-κB activity, TIFA supports tumor cell survival and growth. These data position TIFA not only as a potential prognostic biomarker but also as a candidate therapeutic target in lung adenocarcinoma.

### 4.3 Hematologic malignancies

#### 4.3.1 Acute myeloid leukemia

TIFA exhibits context-dependent functional diversity in hematologic malignancies, acting predominantly as a tumor promoter in acute myeloid leukemia (AML). In *de novo* AML, Aurora A directly phosphorylates TIFA at Thr9, a modification that is essential for NF-κB activation and subsequent leukemic progression. Through this Aurora A-dependent NF-kB signaling axis, TIFA sustains the expression of NF-κB-dependent anti-apoptotic factors, including Bcl-2 and Bcl-XL, thereby promoting leukemic cell proliferation and chemoresistance (12). Functional studies demonstrate that siRNA-mediated silencing of TIFA markedly suppresses growth of AML cell lines and primary patient-derived blasts while enhancing chemosensitivity. Furthermore, *in vivo* delivery of TIFA inhibitory peptides enhances chemotherapeutic efficacy and facilitates leukemic cell clearance in xenograft models, underscoring the therapeutic potential of targeting TIFA in AML.

In AML, TIFA links genotoxic stress to NF-κB activation by cooperating with TRAF2 to promote NEMO ubiquitination, thereby enhancing the expression of pro-survival inflammatory genes and supporting leukemia cell survival (33).

#### 4.3.2 Multiple myeloma

It is noteworthy that the role of TIFA in hematologic malignancies is not uniform. In multiple myeloma cells, TIFA has been reported to undergo nuclear translocation and accumulate on damaged chromatin in a Thr9 phosphorylation-dependent manner. In this DNA damage-associated context, TIFA cooperates with TRAF2, rather than the more commonly described TRAF6, to promote NEMO ubiquitination and downstream NF-κB activation (33).

Collectively, these findings highlight the context-dependent duality of TIFA signaling in hematologic cancers, functioning either as an oncogenic driver or a potential growth suppressor depending on the disease landscape and signaling environment.

### 4.4 Colorectal cancer

Colorectal cancer (CRC) remains one of the most prevalent malignancies worldwide, ranking third in incidence and second in cancer-related mortality. In recent years, the association between gut microbiota and the occurrence and development of CRC has received widespread attention, among which the TIFA-mediated inflammatory signaling pathway has gradually become a research hotspot (45).

Recent studies demonstrate that *Fusobacterium nucleatum* promotes colorectal tumorigenesis through the release of the bacterial metabolite ADP-heptose, which activates the host ALPK1–TIFA signaling axis. Upon entry into the tumor microenvironment, ADP-heptose engages the ALPK1/TIFA/TRAF6 pathway in intestinal epithelial cells, leading to robust NF-κB activation and promoting the expression of inflammation-related genes: upregulating IL-8, BIRC3, TNFAIP3, promoting cell survival, and reducing 5-fluorouracil chemosensitivity. This mechanism provides molecular insight into how microbial metabolites exploit TIFA-dependent signaling to couple intestinal inflammation with oncogenic processes.

Clinically, TIFA expression is significantly elevated in CRC tissues compared with adjacent normal mucosa and positively correlates with TNM stage. Functional studies indicate that TIFA depletion markedly suppresses tumor cell proliferation without substantially affecting apoptosis, whereas ectopic overexpression enhances proliferative capacity both *in vitro* and *in vivo*. Importantly, mutation of the oligomerization site (T9A) or deletion of the TRAF6-interacting domain (D6) abrogates TIFA-driven proliferative effects, underscoring the requirement of TIFA oligomerization and TRAF6 engagement for its oncogenic activity. Mechanistically, TIFA promotes CRC progression through activation of RSK and the mTOR pathway regulator PRAS40, thereby enhancing protein synthesis, cellular growth, and metabolic reprogramming (46).

Collectively, these findings position TIFA as a central signaling integrator that links microbial metabolite sensing to inflammatory amplification and tumor cell proliferation, functioning as a pivotal mediator in colorectal cancer progression.

### 4.5 Hepatocellular carcinoma

In contrast to its tumor-promoting roles in several solid tumors, TIFA appears to exert a tumor-suppressive function in hepatocellular carcinoma (HCC). TIFA expression is downregulated in HCC tissues and cell lines, and its restoration inhibits hepatoma cell proliferation by inducing apoptosis and cell cycle arrest. Mechanistically, TIFA overexpression in HCC cells leads to robust activation of the JNK and p38 MAPK pathways. JNK activation primarily mediates TIFA-induced apoptosis, whereas p38 promotes cell cycle arrest through modulation of the p53-p21 signaling axis. In addition, TIFA overexpression triggers activation of Caspase-8 and Caspase-3, thereby engaging a canonical caspase-dependent apoptotic cascade (47).

Further investigations reveal that the tumor-suppressive effects of TIFA are dependent on its interaction with TRAF6. The C-terminal amino acid residues 178–184 of TIFA constitute the critical binding region required for TRAF6 association. Through TRAF6-mediated signal transduction, sustained TIFA overexpression results in persistent activation of JNK and p38 signaling, thereby inducing apoptosis and suppressing tumor growth in both *in vitro* and *in vivo* models (48). Concurrently, TIFA upregulates p21 expression, reinforcing cell cycle blockade and further restraining hepatocellular carcinoma cell proliferation.

This HCC-specific phenotype suggests that TIFA function is highly context dependent. In contrast to settings in which TIFA may preferentially engage pro-survival or pro-inflammatory outputs, HCC appears to favor TRAF6-dependent stress signaling over TRAF2-associated survival signaling, thereby shifting the downstream consequence toward apoptosis and growth arrest rather than NF-κB-driven tumor promotion. In addition, differences in subcellular assembly of TIFA-containing signaling complexes and the distinctive immunometabolic environment of the liver may further bias TIFA signaling toward JNK/p38–p53–p21 activation and caspase-dependent cell death.

Thus, although TIFA can function as a tumor suppressor in both HCC and MM, the upstream adaptor usage, subcellular deployment, and downstream biological consequences appear to be shaped by lineage-specific signaling architecture rather than by TIFA itself acting through a uniform mechanism.

### 4.6 Cardiovascular diseases

#### 4.6.1 Acute myocardial infarction

TIFA plays a critical regulatory role in the inflammatory response and post-infarction cardiac remodeling following acute myocardial infarction (AMI). And NF-κB plays an important role in cardiovascular diseases (49). Experimental studies using murine AMI models have demonstrated a marked upregulation of TIFA expression after myocardial injury, accompanied by significantly elevated levels of pro-inflammatory cytokines, including interleukin-1β (IL-1β) and tumor necrosis factor-α (TNF-α). These findings suggest that TIFA contributes to the inflammatory cascade triggered by ischemic myocardial damage. TIFA knockdown attenuates inflammatory cell infiltration and suppresses the production of IL-1β, TNF-α, and other pro-inflammatory mediators. This anti-inflammatory effect is largely associated with reduced activation of the NF-κB signaling pathway. In addition, TIFA silencing decreases the expression of matrix metalloproteinase-9 (MMP-9), a key enzyme involved in extracellular matrix degradation and adverse cardiac remodeling, thereby mitigating structural remodeling of the infarcted myocardium (50).

Mechanistically, TIFA-mediated NF-κB activation represents a central molecular event underlying inflammation amplification and myocardial remodeling after AMI. Inhibition of TIFA partially improves cardiac remodeling through suppression of NF-κB signaling. Moreover, TIFA inhibition has been associated with increased apoptosis and cell cycle arrest, indicating a complex regulatory role in post-infarction cellular responses.

Collectively, these findings indicate that TIFA functions as a pivotal modulator of NF-κB-dependent inflammatory signaling and extracellular matrix remodeling in the setting of AMI. Targeting TIFA may therefore represent a potential therapeutic strategy for preventing adverse cardiac remodeling and improving outcomes following myocardial injury.

#### 4.6.2 Pulmonary arterial hypertension

PAH is a fatal disease characterized by pulmonary vascular remodeling and progressive elevation of pulmonary vascular resistance, and its pathogenesis is closely related to chronic inflammation. In recent years, TIFA, as an inflammatory signaling regulatory molecule, has gradually attracted attention for its potential role in PAH.

Chang HC and colleagues found that TIFA protein expression in the peripheral blood of PAH patients was significantly increased. TIFA protein expression can independently predict the presence of PAH and outperforms traditional echocardiographic estimation. It was ultimately found that IL-1β and TNF-α mediated 80.4% and 56.6% of the causal relationship between TIFA and PAH (51). It also suggests the idea that TIFA protein is involved in the pathogenesis of PAH and may have potential value as a biomarker and therapeutic target.

#### 4.6.3 Atherosclerosis

TIFA has emerged as an important mediator linking oxidative stress to vascular inflammation in atherosclerosis. In endothelial cells exposed to atheroprone flow, oxLDL, or TNF-α, TIFA expression is significantly upregulated and contributes to sustained innate immune activation. Mechanistically, TIFA participates in both priming and activation of the NLRP3 inflammasome. SREBP2-driven transcriptional induction of TIFA facilitates signal 1, enhancing inflammatory gene expression, whereas AKT-dependent phosphorylation of TIFA at Thr9 promotes its oligomerization and interaction with caspase-1, thereby facilitating higher-order assembly of the NLRP3 inflammasome (signal 2). This dual regulatory role positions TIFA as a critical amplifier of endothelial inflammation and suggests that persistent TIFA activation may contribute to the chronic inflammatory milieu characteristic of atherosclerotic lesions (13).

Consequently, targeting TIFA may represent a potential therapeutic strategy for vascular inflammatory diseases.

### 4.7 Acute kidney injury

Acute kidney injury (AKI) is one of the most common complications of sepsis and is associated with poor prognosis. Globally, sepsis remains a leading cause of infection-related mortality. In China, the annual incidence reaches several million cases, of which approximately 50% of sepsis patients may progress to sepsis-associated acute kidney injury (SA-AKI). Dysregulated inflammation, mitochondrial dysfunction, and programmed cell death are regarded as the central mechanisms underlying the initiation and progression of SA-AKI. In recent years, TIFA (TRAF-interacting protein with FHA domain) has gradually attracted increasing attention in this context.

A study by Li Y et al. demonstrated that TIFA and fatty acid synthase (FASN) are differentially expressed in the urine of patients with SA-AKI, suggesting their potential value as biomarkers. Tissue localization studies further indicated that TIFA is predominantly expressed in Lotus tetragonolobus lectin (LTL)-positive proximal tubular epithelial cells in the kidney, implying its involvement in the process of tubular injury. Mechanistic investigations have shown that TIFA contributes to tubular epithelial cell dysfunction by regulating mitochondrial injury and is closely associated with pyroptosis. TIFA activates inflammatory signaling pathways related to mitochondrial damage, thereby exacerbating cellular injury (52).

Additionally, METTL3 enhances the stability of TIFA mRNA through m^6^A modification, while the m^6^A reader protein IGF2BP2 further promotes its expression. Upregulated TIFA activates the NF-κB signaling pathway, facilitates NLRP3 inflammasome assembly and Caspase-1 activation, and induces GSDMD cleavage as well as IL-1β and IL-18 release, thereby driving pyroptosis in renal tubular epithelial cells (53).

Overall, TIFA not only participates in inflammatory amplification and mitochondrial injury in sepsis-associated acute kidney injury but may also modulate NF-κB-dependent cell death signaling. Targeting TIFA can ameliorate inflammatory responses and alleviate renal dysfunction, highlighting its potential as a therapeutic target for SA-AKI.

### 4.8 Ischemic stroke

Ischemic stroke is a leading cause of disability and mortality worldwide, and its pathogenesis is closely associated with sterile inflammation following acute cerebral ischemia. In recent years, the ALPK1-TIFA signaling axis has attracted increasing attention in ischemic brain injury.

In ischemia–reperfusion-associated injury, TIFA acts more as an inflammatory amplifier than as a primary initiating sensor. Its upregulation strengthens TLR4/MyD88/NF-κB signaling and is associated with HMGB1-dependent feed-forward loops, thereby prolonging sterile inflammation and tissue damage. Knockdown of ALPK1 inhibited TIFA phosphorylation, reduced TRAF6 expression, suppressed NF-κB activation, and decreased pro-inflammatory cytokine production in BV2 microglial cells. Furthermore, by suppressing the TIFA/TRAF6/NF-κB signaling pathway and attenuating neuroinflammation in mice, ALPK1 knockdown conferred neuroprotection against ischemic brain injury (54).

Current evidence indicates that ALPK1 is involved in sterile inflammatory injury following acute cerebral ischemia, providing preliminary support for the therapeutic potential of targeting ALPK1 in ischemic stroke.

### 4.9 Newcastle disease virus

Mutation of the nuclear localization signal (NLS) within the matrix (M) protein of Newcastle disease virus (NDV) not only disrupts the nucleocytoplasmic trafficking of M, but also impairs viral RNA synthesis and transcription. Duan Z. et al. further demonstrated that, in the cytoplasm, the M protein suppresses TIFA expression in a dose-dependent manner, thereby facilitating NDV replication through attenuation of TIFA/TRAF6/NF-κB-mediated cytokine production (55).

### 4.10 Scalp psoriasis

**Figure 5:**
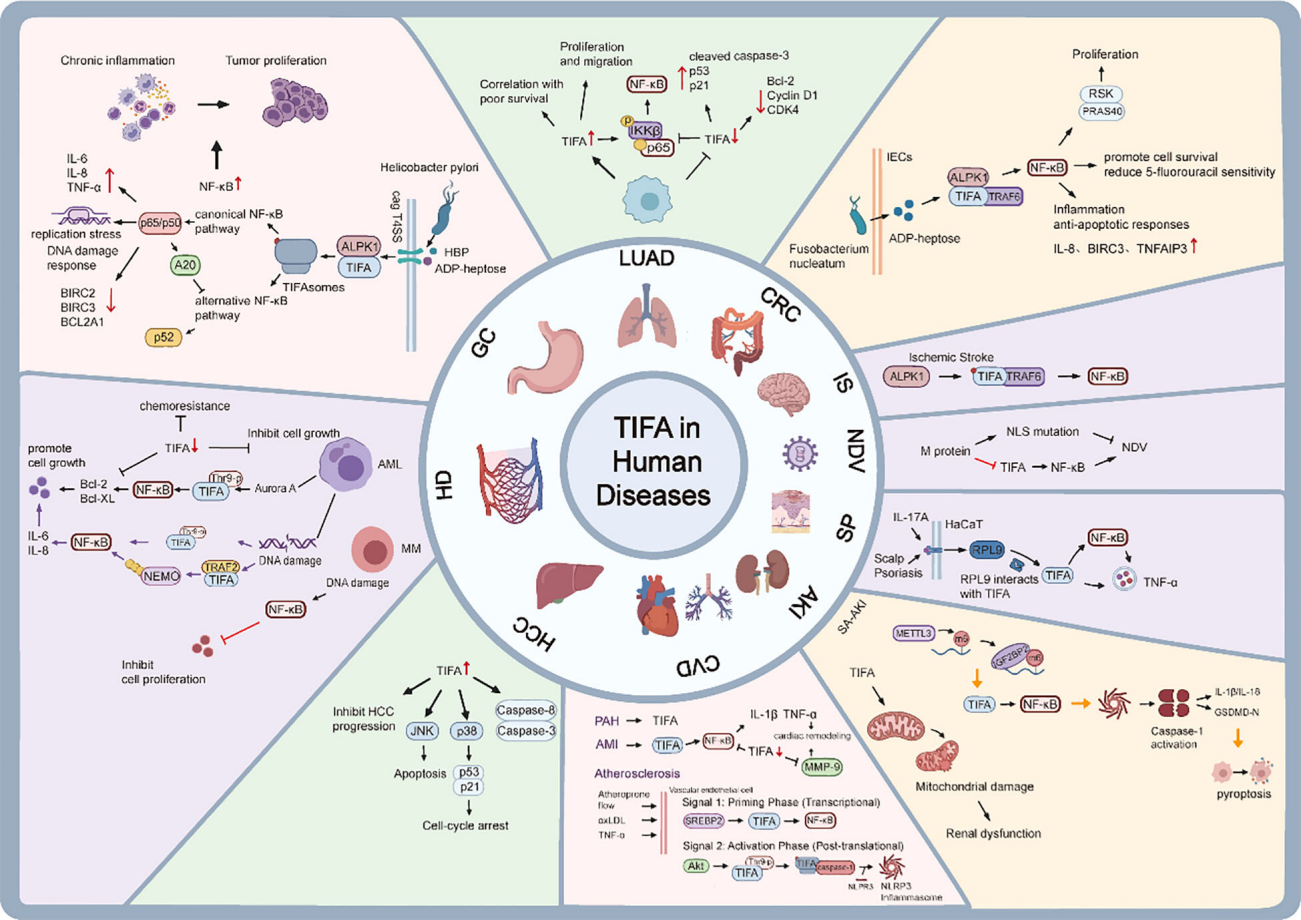
Overview of TIFA functions in human diseases. Schematic summary of the roles and mechanisms of TIFA across multiple human diseases, including gastric cancer (GC), lung adenocarcinoma (LUAD), colorectal cancer (CRC), ischemic stroke (IS), Newcastle disease virus infection (NDV), scalp psoriasis (SP), acute kidney injury (AKI), cardiovascular disease (CVD), hepatocellular carcinoma (HCC), and hematologic disorders (HD). In different pathological contexts, TIFA regulates inflammation, NF-κB signaling, apoptosis, pyroptosis, cell-cycle progression, DNA damage responses, and tumor growth through interactions with upstream factors such as ALPK1, ADP-heptose, microbial products, and disease-associated signaling molecules. The image highlights the context-dependent functions of TIFA in promoting or suppressing disease progression and summarizes representative downstream pathways and effectors associated with each condition.

Experimental evidence has demonstrated that both RPL9 and TIFA are highly expressed in lesional tissues from scalp psoriasis and in IL-17A-stimulated HaCaT cells. Liu S. et al. further reported that RPL9 interacts with TIFA in IL-17A-treated HaCaT cells. qPCR and western blotting analyses showed that RPL9 knockdown markedly reduced both the mRNA and protein levels of TIFA in HaCaT cells. In addition, ELISA results revealed that silencing of RPL9 significantly suppressed TNF-α secretion in IL-17A-stimulated HaCaT cells (56) (**Figure 5**).

## 5 Therapeutic targeting of TIFA: preclinical approaches and translational challenges

At present, direct therapeutic targeting of TIFA remains largely unexplored. No validated small molecules, peptides, or antibodies specifically directed against TIFA have been reported, likely reflecting the intracellular adaptor nature of TIFA and the limited structural information available for drug development. In contrast, genetic perturbation strategies, including RNA interference, mutagenesis, and pathway-level gene manipulation, have been widely used as research tools to define TIFA function in innate immunity, inflammation, and cancer. These studies suggest that future intervention strategies may focus on disrupting the TIFA FHA–pThr9 interface, blocking TIFA oligomerization, or modulating upstream kinases such as ALPK1 and AKT. At present, genetic and nucleic acid-based strategies, including siRNA, shRNA, antisense oligonucleotides, and CRISPR/Cas approaches, represent the most practical.

From a translational perspective, TIFA-targeted therapy is still at an early stage, and current preclinical strategies are more likely to rely on indirect approaches such as RNA-mediated knockdown, TIFAB restoration, or disruption of the TIFA FHA-pThr9 interaction. However, clinical translation remains challenging because TIFA is an intracellular adaptor with limited targeted drugs, its function is highly dependent on the specific context, and it requires delivery to specific cells. Therefore, although TIFA is a promising therapeutic target, successful clinical translation may require precise patient stratification, biomarker-based targeting, and tissue-specific therapeutic delivery.

## 6 Discussion and future perspectives

Collectively, current evidence supports TIFA as a context-dependent signaling integrator rather than a uniformly pro-tumorigenic or tumor-suppressive factor. Although TIFA is frequently linked to NF-κB activation, inflammatory amplification, and tumor cell survival, this model does not fully explain why TIFA promotes proliferation in some malignancies but restrains growth or enhances apoptosis in others. These divergent effects likely reflect differences in upstream stimuli, TRAF partner usage (especially TRAF6 versus TRAF2), subcellular localization, and the status of tumor suppressive pathways such as p53. In lung adenocarcinoma and colorectal cancer, chronic exposure to cytokines, microbial products, or danger signals may favor TRAF6-dependent NF-κB and growth-related pathways, thereby promoting proliferation, survival, and inflammatory remodeling; in colorectal cancer, microbial metabolite-driven signaling may further connect intestinal inflammation to oncogenic growth. In contrast, in hepatocellular carcinoma, the liver microenvironment is shaped by metabolic stress, chronic hepatitis, cirrhosis, immune tolerance, and repeated injury–repair cycles, under which TIFA may be more closely linked to stress signaling, p53/p21-mediated cell-cycle arrest, and apoptosis, thus exerting tumor-suppressive effects. In addition, differential TRAF partner usage and the status of tumor-suppressive pathways may further influence TIFA function, such that TIFA tends to support survival in tumors with dominant NF-κB/mTOR activity or defective p53 signaling, but may promote growth arrest or apoptosis in cells with intact p53/p21 or stress kinase responses.

Finally, TIFA function is likely shaped not only by activation mechanisms but also by signal termination and protein turnover. Negative regulators such as TIFAB, ubiquitin-editing enzymes, and proteasomal or lysosomal degradation pathways may determine the duration and intensity of TIFA signaling. Insufficient clearance of activated TIFA complexes could sustain NF-κB activation and promote chronic inflammation, whereas efficient turnover or redirection toward stress-response pathways may limit tumor growth. These regulatory layers remain incompletely understood and should be systematically investigated.

Therefore, future research should focus more on targeted therapy and translational medicine to determine whether ALPK1 inhibitors, TIFA oligomerization blockers, or strategies targeting the TIFA-TRAF interaction can provide therapeutic benefits with acceptable specificity and safety in specific disease contexts.

In summary, TIFA is regarded as a multifunctional signaling hub at the intersection of innate immunity, inflammation, stress responses, and cancer, and studying the mechanisms of its functions in specific contexts is crucial for determining when TIFA acts as a pathogenic driver and when it plays a protective role.

## Glossary

PAMPs: Pathogen-Associated Molecular Patterns
PRRs: Pattern Recognition Receptors
TIFA: TRAF-Interacting protein with a Forkhead-associated domain
FHA: Forkhead-associated
TNFR: Tumor Necrosis Factor Receptor
TLR: Toll-like receptor
Glu178: Glutamic acid at position 178
pThr: pT, Phosphorylated Threonine
TRAF6: Tumor Necrosis Factor Receptor–Associated Factor 6
Glu178: Glutamic acid at position 178
LSD: Lysine-specific demethylase
HMGB1: High-mobility group box 1
ADP-heptose: ADP-β-D-manno-heptose
NIK: NF-κB-inducing kinase
R-loops: RNA/DNA hybrids
CsrA: Carbon storage regulator A
LUAD: Lung Adenocarcinoma
AML: Acute Myeloid Leukemia
CRC: Colorectal Cancer
HCC: Hepatocellular Carcinoma
AMI: Acute Myocardial Infarction
TNF-α: Tumor Necrosis Factor-α
MMP-9: Matrix MetalloProteinase-9
PAH: Pulmonary Arterial Hypertension
AKI: Acute Kidney Injury
SA-AKI: Sepsis-Associated Acute Kidney Injury
FASN: Fatty Acid Synthase
LTL: Lotus Tetragonolobus Lectin
ALPK1: Alpha-Protein Kinase 1
NLS: Nuclear Localization Signal
NDV: Newcastle Disease Virus
GSDMD: Gasdermin D
BIRC3: Baculoviral IAP Repeat Containing 3
A20/TNFAIP3: Tumor Necrosis Factor alpha-Induced Protein 3
AKT: Protein Kinase B
cIAP1: Cellular Inhibitor of Apoptosis Protein 1
IKK: IκB Kinase
IL-1: Interleukin-1
IL-6: Interleukin-6
IL-8: Interleukin-8
IRAK1: Interleukin-1 Receptor-Associated Kinase 1
JNK: c-Jun N-terminal Kinase
K63: Lys63-linked ubiquitin
MAPK: Mitogen-Activated Protein Kinase
METTL3: Methyltransferase-like 3
MyD88: Myeloid Differentiation primary response 88
NEMO: NF-κB Essential Modulator
PI3K: Phosphoinositide 3-Kinase
TLR4: Toll-like Receptor 4
TRAF2: TNF Receptor-Associated Factor 2
TAK1: Transforming Growth Factor-β-Activated Kinase1.
